# Idiopathic intracranial hypertension in child

**DOI:** 10.22336/rjo.2020.66

**Published:** 2020

**Authors:** Mihail Zemba, Andreea-Elena Dimirache, Roxana-Elena Rogoz

**Affiliations:** * Ophthalmology Department, “Dr. Carol Davila” Central Military University Emergency Hospital, Bucharest, Romania; ** “Carol Davila” University of Medicine and Pharmacy, Bucharest, Romania

**Keywords:** papilledema, idiopathic intracranial hypertension, pseudotumor cerebri

## Abstract

We present the case of a 12-year-old boy with bilateral papilledema, relating moderate symptomatology and without an important medical history. Ophthalmological examination revealed a significant alteration of visual acuity, an important papilledema and macular edema in both eyes. Further investigations excluded infectious and autoimmune diseases, intracranial masses and congenital affliction. Because of an elevated opening pressure in lumbar puncture procedure, the diagnosis of intracranial hypertension was confirmed. After two weeks of treatment with corticosteroids, carbonic anhydrase inhibitor and hyperosmotic drug, the patient had an important structural and functional ophthalmological improvement.

## Introduction

Intracranial hypertension is a condition due to high pressure of the cerebrospinal fluid within the spaces that surround the brain and the spinal cord [**1**]. It can be either secondary to cerebrospinal and vascular pathology or idiopathic. 

Idiopathic intracranial hypertension (IIH), also known as pseudotumor cerebri, is a syndrome related to elevated intracranial pressure of unknown cause, which implies the absence of intracranial mass lesions or clear cerebrospinal fluid outflow obstruction. IIH occurs most commonly in obese women of reproductive age [**2**]. 

The incidence is approximately 1 /100 000/ year in the general population, but is much more common in obese women of childbearing age. Thus, the incidence rises to 13/ 100 000/ year in women aged 20-44 who are 10% above the ideal body weight and 19/ 100 000/ year in those 20% above the ideal body weight [**3**]. 

Various medications including high-dose vitamin A derivates (>100.000 U/ day), contraceptive pills, growth hormones, tetracycline, doxycycline, retinoic acid, indomethacin, ketoprofen, minocycline, corticosteroids, levothyroxine and lithium have been implicated. Systemic conditions such as hypertension, anemia, thyroid disease, lupus erythematosus, Lyme disease, sleep apnea syndrome, Addison, Cushing, multiple sclerosis are related to IIH [**4**].

The signs and symptoms of intracranial hypertension usually appear in an otherwise alert and oriented patient, with normal mental state and no localized neurological findings [**5**]. Most of the patients present with headache as a prominent feature. The headache phenotype is progressive, starting retro-orbitally, worsening with eye and head movement, bending, coughing and with clinostatism. The pain may be localized or generalized. Other symptoms of IIH may include nausea, occasional projectile vomiting, pulsatile tinnitus and dizziness, which may progress to deterioration of consciousness as severity increases. 

Visual symptoms, which appear in 80% of the cases: blurred vision or metamorphopsia of central vision, dyschromatopsia, photopsia, visual obscuration affecting one or both eyes, horizontal diplopia due to sixth nerve palsy; visual acuity is usually normal or minimally reduced; significant reduction is a late feature in conjunction with secondary optic atrophy [**6**,**7**].

The Modified Dandy criteria comprise clinical, laboratory and radiological findings required for a diagnosis of IIH. The criteria required are: 1) symptoms and sings of increased intracranial pressure (e.g. papilledema and headache), 2) CSF pressure >250 mm of water in the lateral decubitus position, 3) no localization signs except for sixth nerve palsy, 4) normal CSF composition, 5) normal-to-small ventricles on imaging with no intracranial mass, 6) no unexplained symptoms and signs, 7) exclusion of other causes on specific forms of imaging in, particular, MRI/ venography should be included to rule out intracranial venous sinus thrombosis [**8**].

Ocular manifestations appear as a result of 2 main pathogenic mechanisms: 1) mechanical component that determines the following changes: blurring of the optic disc margins, filling in of the optic disc cup, anterior extension of the optic nerve head, edema of the nerve fiber layer and retinal/ choroidal folds; 2) vascular component: venous congestion of arcuate and peripapillary vessels, papillary and retinal peripapillary hemorrhages, nerve fiber layer infarcts, hyperemia of the optic nerve head, hard exudates of the optic disc [**9**].

## Case report

We present the case of a 12-year-old male who came to our emergency department complaining of progressive visual loss and intermittent frontal headache of moderate intensity, not accompanied by photophobia or ocular pain. 

Symptoms started 2 weeks before, and had a progressively worsening course over the next days. Associated symptoms included apathy, accentuated in the last 3 days, nausea and 2 episodes of vomiting. He had no history of migraine headaches or any recent illness or fever. His medical records showed several episodes of recurrent laryngitis in the past.

The general examination was within normal limits, except for the blood pressure, which was 140/ 102+ mmHg. His weight was 81 kg, height 170 cm and BMI 28 kg/ m2.

On clinical examination, BCVA was 20/ 120 in the right eye and 20/ 200 in the left eye. The patient also presented a mild red-green color vision defect. Non-contact tonometry indicated an IOP value of 20 mmHg in both eyes. The patient had normal function of the cranial nerves III, V, VI and VII bilaterally. 

**Fig. 1 A,B:**
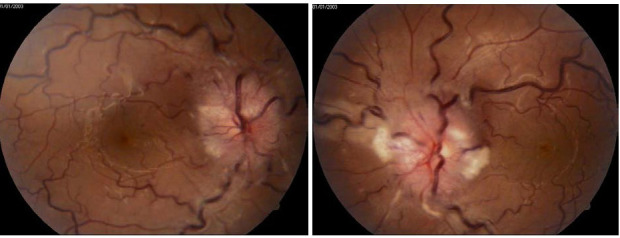
Fundus photos showing an intense hyperemic optic disc with blurred margins, venous congestion and choroidal folds

Slit-lamp examination revealed a normal appearance of the anterior segment in both eyes. On fundus examination, an anomalous optic disc was noted with similar aspect in both eyes. Abnormal findings consisted of an elevated and highly hyperemic optic disc with blurred margins and filling the optic disc cup. Moreover, retinal and choroidal folds were present. Vascular changes included venous congestion of arcuate and peripapillary vessels, flame-shaped hemorrhages, cotton wool spots and hard exudates (**Fig. 1A, B**). Macular examination showed no apparent changes.

**Fig. 2 A,B:**
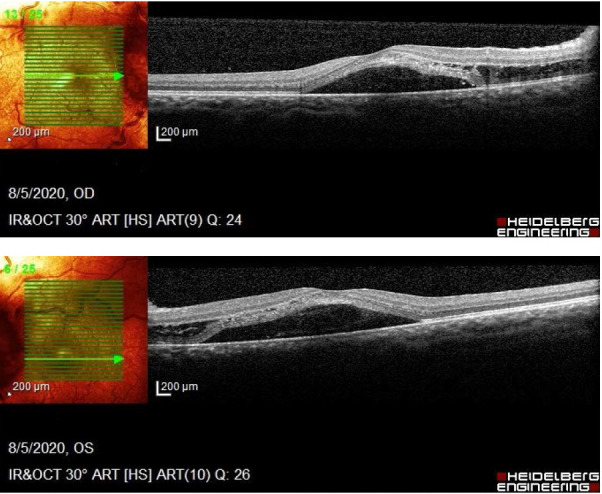
Macular OCT examination shows subretinal liquid deforming the foveal region in both eyes

An Optical Coherence Tomography was performed in order to assess the degree of severity of retinal damage. Macular examination revealed deformation of the foveal contour in both eyes and increased retinal thickness due to the accumulation of intraretinal fluid (**Fig. 2A, B**). On tomographic examination of the optic nerve, changes included an artefactual increase of the thickness of the retinal nerve fiber layer (RNFL), a blurred margin of the papilla and a significant decrease of the topographic parameters of the excavation.

**Fig. 3 A,B:**
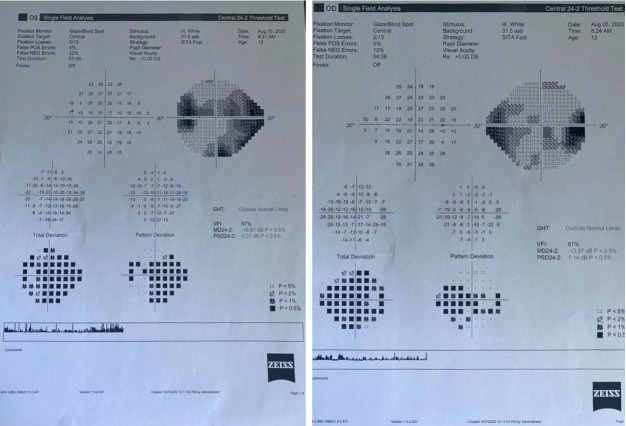
Perimetry of the right and left eye showing a blind spot enlargement and unpatterned visual field loss

Humphrey Visual Field Analyzer perimetry was attempted and showed a mean deviation of -13.37 dB in the right eye with enlargement of the blind spot and a dense nasal field defect. In the left eye, the mean deviation was -16.81 dB and also showed an enlargement of the blind spot and a dense nasal field defect (**Fig. 3A, B**). Also, both eyes presented unpatterned reduction of the light sensitivity.

An immunologic screening demonstrated negative anti-nuclear antibodies and anti-dsDNA in normal limits. A serologic infectious screening was performed and was negative for HIV infection, borreliosis, cat scratch disease, listeriosis and toxoplasmosis.

When he presented to our clinic, the patient had already undergone an MRI that showed mild ventriculomegaly with no apparent obstacle in the drainage of CSF. 

We suspected the diagnoses of idiopathic intracranial hypertension and referred the patient to a pediatric neurologist for further workup and management.

In the first day of hospitalization in the pediatric neurology department, the patient developed diplopia due to abducens nerve palsy.

Cerebral angio MRI showed: mild internal tetraventricular hydrocephalus, periventricular white matter macrostructure alteration, a complete configuration of the circle of Wills without any cerebral arteriovenous malformation, a selar and orbital region with normal MRI signal; hypoplasia of the right transverse sinus.

The patient was put on a low-calorie diet. Mannitol 150 mg/ ml for 10 days was initiated, associated with Dexamethasone 8 mg/ 2 ml injection for 10 days, the latter being subsequently switched to Methylprednisolone 16 mg for 4 days. 

The neurologist performed a lumbar puncture that showed an elevated opening pressure of 25 mmHg (34 cm H2O) and thus confirmed the diagnosis of intracranial hypertension. Subsequently, Acetazolamide 250 mg was added to the treatment in progressive highly dosage, from 250 mg/ day to 1000 mg/ day.

After 14 days of hospitalization, the patient was discharged with the following treatment: Methylprednisolone, Acetazolamide and proton-pump inhibitors.

We reevaluated the patient after his hospital discharge. BCVA was significantly improved, 20/ 20 in both eyes and normal ocular motility in both eyes with no diplopia.

**Fig. 4 A,B:**
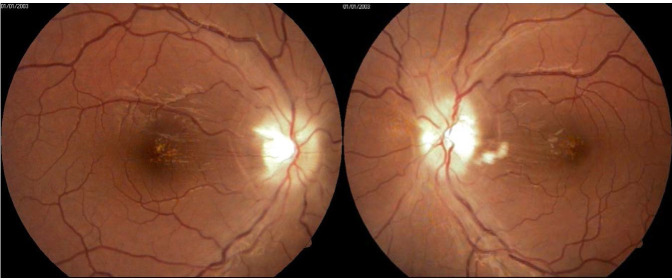
Fundus examination of both eyes - blurred disc margins and macular hard exudates

Slit-lamp examination revealed a normal appearance of the anterior segment in both eyes. On fundus examination, we identified a significant reduction of the papillary edema, blurred margins of the optic disc but no elevation of the disc head and hard exudates in the foveolar and parafoveolar area. Also, the vascular configuration and calibre improved (**Fig. 4A, B**).

**Fig. 5 A,B:**
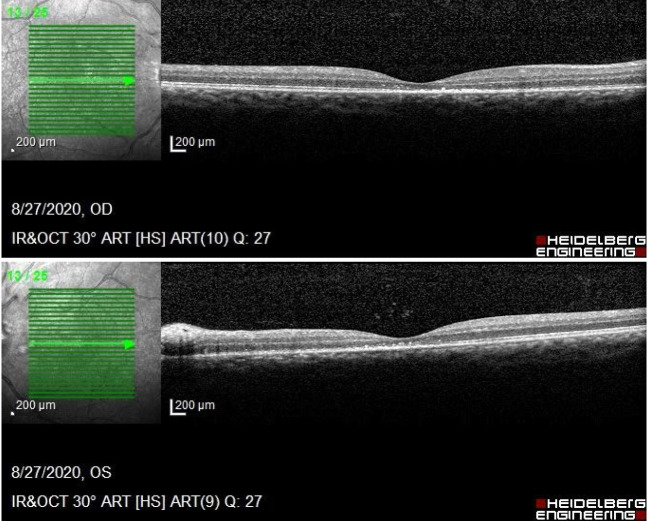
Macular OCT of both eyes after treatment - hyperreflective dots in the macular area

**Fig. 6 A,B:**
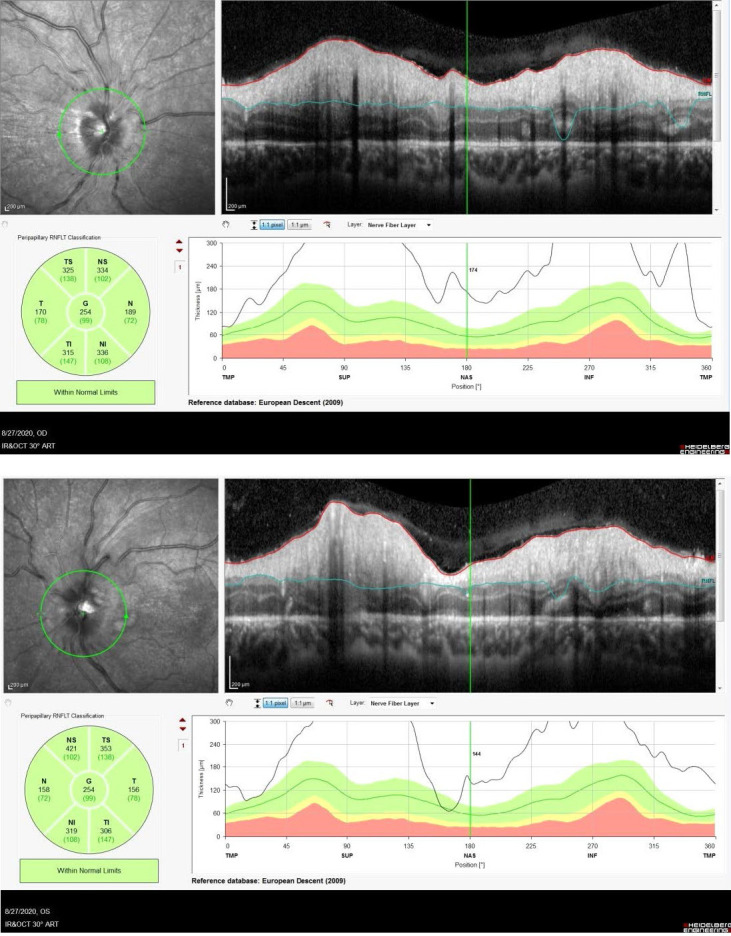
Follow up OCT examination - remnant edema of the optic disc in both eye

An Optical Coherence Tomography was performed to assess the degree of severity of retinal damage. Macular examination showed no subretinal liquid, retinal thickness returned to normal values and hard exudates (hyperreflective dots) were present in the macular area (**Fig. 5A, B**). On tomographic examination of the optic nerve, the average thickness of the retinal nerve fiber layer (RNFL) in both eyes was still above normal limits, with slightly blurring of the optic disc margins (**Fig. 6A, B**).

**Fig. 7 A,B:**
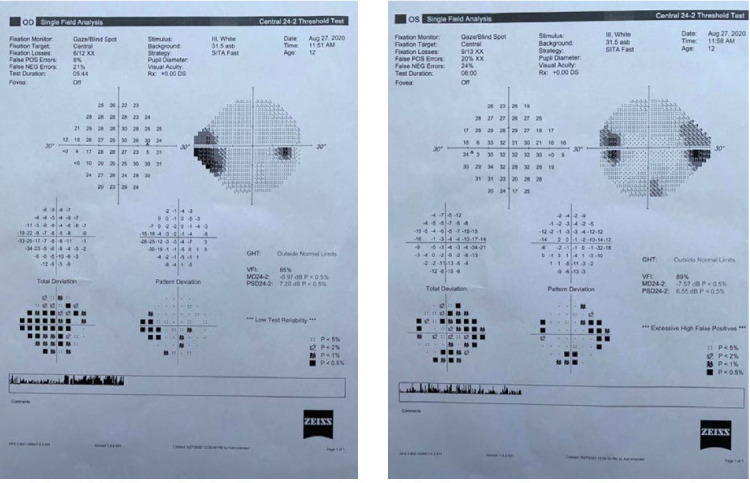
Visual field with remnant defects in both eyes and normal blind spot size

On visual field examination, a significant improvement was noted: a mean deviation of -8.97 dB in the right eye with normal blind spot size and the persistence of a nasal defect. In the left eye, the mean deviation was -7.57 dB with some residual nasal and temporal defects (**Fig. 7A, B**).

## Discussions

Idiopathic intracranial hypertension (IIH) denotes a raised intracranial pressure occurring in the absence of intracranial mass lesions and without impairment of the cerebrospinal fluid outflow.

The differential diagnosis should include a large spectrum of underlying diseases (intracranial mass lesions, hydrocephalus, cerebral venous thrombosis, etc.). Children presenting with papilledema require a prompt evaluation with cerebral imaging to rule out an intracranial tumor or a cerebral venous thrombosis. After excluding a space occupying lesion, primary benign intracranial hypertension is an important differential diagnosis [**10**]. 

 Our patient met the Modified Dandy Criteria for IIH. Firstly, he presented with symptoms of intracranial hypertension, such as headaches, nausea with projectile vomiting and papillary edema. Secondly, the CSF pressure on lumbar puncture was 34 cm H2O, above the 25 cm H2O limit. Thirdly, apart from a VI nerve palsy that he developed during his admission in the pediatric neurology department, no other localized neurological sign was identified. 

What is more, the patient underwent both MRI and angio MRI investigations, that ruled out any space-occupying cranial lesions as well as cavernous sinus thrombosis, both of which could have been possible causes. Mild internal tetraventricular hydrocephalus which, in conjunction with the lack of CSF outflow obstruction, supported the diagnosis of IIH.

Another supporting element was the prompt response to treatment, which consisted of mannitol, dexamethasone and acetazolamide. These medications reduced the intracranial pressure and, consequently and fairly rapidly, the patient regained a normal visual function, with a BCVA improving from 20/ 120 in the right eye and 20/ 200 in the left to 20/ 20 in both eyes during the course of just two weeks. Moreover, fundoscopic examination showed improvement in vascular caliber and configuration, reduction of papillary edema, while the OCT demonstrated the disappearance of the submacular fluid.

The particularity of our case resided in two aspects: the epidemiology and the presentation.

Epidemiologically, IIH has a major predilection for a populational group comprising obese women of childbearing age. The disease is rare in prepuberty children, in whom obesity is less common [**3**]. Our patient was a 12-year-old boy, so not in the target age or group. However, he had the weight risk factor, with a BMI of 28 kg/ m2. 

In a study including 1058 obese children and adolescents between 2 and 18 years of age, researchers studied the prevalence of IIH and associated factors. They diagnosed 14 (1.32%) subjects with IIH, the male-female ratio being 2/ 5. Moreover, complaints of headache, blurred vision and vomiting were significantly higher in subjects with IIH compared to other subjects. In this study, insulin resistance, dyslipidemia and hepatosteatosis were found to be more prevalent with IIH than the others [**11**]. Our patient’s abnormal blood tests that could suggest a developing metabolic syndrome, with TGO and TGP levels higher than normal, hypertension, but lipid-line investigation did not identify dyslipidemia. Total lipid level increased after the treatment was initiated.

On the other hand, Anat Kesler et al., who conducted a study in a pediatric population of 34 children, concluded that IIH may be associated with drug use, including homeopathic treatments, but they found no other association, including obesity. Our patient did not admit taking any form of medication.

Regarding the other aspect that individualized our case, the patient’s presenting signs and symptoms were atypical. Firstly, the severely decreased BCVA that our patient expressed in both eyes is not a characteristic finding for IIH. Our patient had a significantly reduced BCVA of 20/ 120 in the right eye and 20/ 200 in the left eye. The severity of his visual function loss and the presence of intraretinal fluid on OCT examination led us to consider the diagnosis of neuroretinitis in a phase preceding the emergence of a macular star. The macular star, a characteristic finding in neuroretinitis, is the result of lipid deposition within the retinal layers after edema remission and usually occurs two weeks in the evolution of neuroretinitis. When the patient presented to our clinic, it was too early to exclude the diagnosis of neuroretinitis based on the absence of the macular star. 

A retrospective analysis of 24 patients with papilledema, showed macular changes in 21 of 48 (44%) eyes. These were: choroidal folds in 9 eyes; circumferential lines (Paton’s lies) in 4 eyes; nerve fiber layers haemorrhage in 3 eyes; macular stars in 5 eyes; macular edema in 6 eyes; retinal pigment epithelial changes in 4 eyes; subretinal haemorrhage leading to a macular star in 1 eye [**12**,**13**]. Researchers also suggested that macular changes should be an indicator for the intervention to normalize intracranial pressure.

However, subsequent investigations, especially the lumbar puncture and MRI, helped establish the diagnosis of IIH over that of neuroretinitis. Moreover, at the follow-up visit two weeks later, no macular star was present, while the intraretinal fluid had completely remitted. 

## Conclusion

A case of a 12-year-old boy with bilateral papilledema was presented. The ophthalmological examination revealed a significant alteration of visual acuity, an important papilledema and macular edema in both eyes. After two weeks of treatment with corticosteroids, carbonic anhydrase inhibitor and hyperosmotic drug, the patient had an important structural and functional ophthalmological improvement.

**Conflict of Interest**

The authors state no conflict of interest.

**Informed Consent**

An informed consent was obtained from the patient included in this study.

**Authorization for the use of human subjects**

The research related to human use complies with all the relevant national regulations, institutional policies, is in accordance with the tenets of the Helsinki Declaration, and has been approved by the Ethics Committee of the Ophthalmology Department, “Dr. Carol Davila” Central Military University Emergency Hospital. 

**Acknowledgements**

None.

**Sources of Funding**

None.

**Disclosures**

None.
